# Beyond discrete classifications: a computational approach to the continuum of cognition and behavior in children

**DOI:** 10.1038/s44184-025-00163-5

**Published:** 2025-10-01

**Authors:** Anthony Gagnon, Virginie Gillet, Anne-Sandrine Desautels, Jean-François Lepage, Andrea A. Baccarelli, Jonathan Posner, Maxime Descoteaux, Marie A. Brunet, Larissa Takser

**Affiliations:** 1 Department of Pediatrics, University of Sherbrooke, Sherbrooke, QC Canada; 2 Department of Environmental Health, Harvard T. H. Chan School of Public Health, Boston, MA USA; 3 Department of Psychiatry and Behavioral Sciences, Duke University, Durham, NC USA; 4 Sherbrooke Connectivity Imaging Lab (SCIL), University of Sherbrooke, Sherbrooke, QC Canada

**Keywords:** Paediatric research, Psychiatric disorders, Cognitive neuroscience, Computational neuroscience

## Abstract

Psychiatry is undergoing a shift toward precision medicine, demanding personalized approaches that capture the complexity of cognition and behavior. Here, we introduce a novel referential of four robust, replicable, and generalizable cognitive and behavioral profiles. These were derived from a large pediatric cohort (ABCD: *n* = 10,843) and validated in two independent cohorts (BANDA: *n* = 195 and GESTE: *n* = 271) regrouping children aged 9–17 years. We demonstrate the profiles’ longitudinal stability and consistency with clinical diagnoses in the general population while exposing critical discrepancies across parent-reported, youth-reported, and expert-derived diagnoses. Beyond validation, we showcase the real-world utility of our approach by linking profiles to environmental factors, revealing associations between parental influences and youths’ cognition and behavior. Our fuzzy profiling framework moves beyond discrete classification, offering a powerful tool to refine psychiatric evaluation and intervention. We provide an open-source framework, enabling researchers and clinicians to fast-track implementation and foster a data-driven, domain-based approach to diagnosis.

## Introduction

The recent years in clinical psychiatry have seen a fueled debate surrounding the current classification systems and their inadequacy to capture the complex nature of psychiatric disorders^1^. One of the core issues with current classification systems, such as the Diagnostic and Statistical Manual of Mental Disorders fifth edition (DSM-V), is the inability to handle the known heterogeneity within a single clinical population as well as the overlap with other populations^2^. New initiatives, such as the Research Domain Criteria (RDoC)^3^ and Hierarchical Taxonomy of Psychopathology (HiTOP)^4^, have proposed frameworks shifting from the traditional categorical view to a dimensional and domain-specific approach. Those frameworks rely on fundamental knowledge in multiple areas of basic science or on data-driven methods to generate empirical domains. Even if they are not widely used in clinical practice, they represent the first steps toward precision medicine in psychiatry and highlight the need to move away from categorical frameworks.

Psychiatric disorders are known to be much more complex than originating from a single cause or defective mechanism, urging clinicians to include environmental, lifestyle, and biological factors within the diagnosis process^5^. However, the influence of environmental factors on psychopathology (e.g., psychiatric disorders) is not fully understood primarily due to the inability to capture the complexity of the symptoms’ presentation^6^. A plethora of studies have shown relationships between environmental factors, such as supportive social relations, the experience of discrimination, and general exposure to adversity, with global mental health/psychopathology^7,8^. This is particularly important in the late childhood and early adolescence periods, which represent a critical developmental window for profiling later psychiatric risk^9^. Those periods are characterized by major neurodevelopmental reorganizations, the emergence of higher-order cognitive abilities, and an increasing incidence of internalizing and externalizing symptomatology^10–12^. Therefore, it is not surprising that childhood and adolescence represent the onset of most psychiatric disorders^13^. Those developmental trajectories are vulnerable to environmental factors, which can shape early patterns of risk, making them a crucial focus for advancing personalized approaches in pediatric psychiatry. Previous studies have supported these effects on developmental trajectories. Leveraging the Adolescent Brain Cognitive Development (ABCD) cohort^14^, researchers found that familial and community factors, such as family income, marital status, and school environment, significantly predicted cognitive outcomes in preadolescents^15^. In the same study, behavioral problems in pre-adolescents were mainly predicted by family conflict, severe financial difficulty, sleep problems, and maternal medical conditions^15^. Additional studies in the same population highlighted an intrinsic relationship between the structure of cognitive abilities and behavioral manifestations, suggesting that worse cognitive abilities are associated with higher behavioral symptoms^16,17^. However, those studies were performed in a single cohort, therefore not extending the generalizability of those results to different populations. As in many fields, generalizable results are hard to achieve and require collecting data from multiple independent populations. Modeling cognition and behavior while retaining the ability to compare and generalize results between populations is still an active open question in modern psychiatry, particularly considering the transition toward personalized medicine. This article tackles this challenge head-on and proposes to model cognition and behavior using fuzzy profiling, thereby embracing the continuum of cognition and behavior found in the general population.

In the past decade, a significant corpus of literature has explored extracting profiles from behavioral and psychopathology data in the general population. Indeed, the use of latent class analysis (also named latent profile analysis) has helped uncover profiles showing similar patterns of behavioral symptoms^18^. Although studies used different indicators and/or examined different symptoms or disorders of interest, all studies identified relevant subgroups, with the majority reporting the low behavioral symptoms profile as the largest group^18^. One specific study examined the relationship between cognitive measures and behavioral symptoms subgroups, reporting worse working memory, processing speed, and cognitive/intelligence quotient in the internalizing profile and overall worse cognitive performance in the externalizing and dysregulation profiles^19^. While latent profile analysis deals with uncertainty in the form of probabilities (e.g., the likelihood that an event occurs), it merely reflects the confidence in the discrete classification, not the degree of belonging to each profile. This concept makes latent profile analysis suited for scenarios where there should not be an overlap between profiles. However, this is not the case in cognition and behavior, where individuals can present overlapping characteristics from multiple profiles or diagnosis groups; therefore, we must fully embrace the fuzziness of the natural continuum of symptomatology and capabilities found in the general population.

Here, we aimed to develop a new referential model that represents the continuum of cognition and behavior in the general population, enabling direct comparison and generalization across studies. We leveraged data from three independent pediatric cohorts: the ABCD cohort^14^, the Boston Adolescent Neuroimaging of Depression and Anxiety (BANDA) cohort^20^, and the GESTation and Environment (GESTE) cohort^21^. From all cohorts, we extracted cognitive and behavioral domains central to current efforts in youth-focused precision psychiatry that directly map onto well-established transdiagnostic models of psychiatric vulnerability^3,4^. We applied a data-driven fuzzy clustering algorithm within ABCD to create our referential cognitive and behavioral profiles. We then showcase the generalizability of our referential profiles by extending them to the two remaining validation cohorts (BANDA and GESTE). Fuzzy logic combined with clustering allows the extraction of patterns, namely profiles, from the data while keeping the natural continuum of cognition and behavior. Unlike probabilistic methods, fuzzy clustering is better suited for overlapping profiles where individuals can exhibit shared characteristics. In classical clustering, two similar subjects are often separated into two different groups by being on opposite sides of the clusters’ boundaries. Therefore, they will be considered entirely differently in subsequent analyses. This separation into groups represents a substantial loss of information compared to continuous methods. Our method enables the retention of this information (e.g., the similarity between subjects through the membership values, which preserves the information typically lost in classical clustering when two subjects are similar but clustered separately) while extracting meaningful profiles from the data, making it more suitable for real-world scenarios.

Additionally, we demonstrate the stability of our method over a range of developmental periods by independently reproducing the profiles in subsequent follow-ups within the ABCD cohort. Furthermore, by leveraging graph theory concepts, we demonstrate the profiles’ consistency with clinical diagnoses in all cohorts, showing good-to-great consistency. Then, we demonstrate how the profiles can be used to study pressing real-world research questions by investigating the impact of environmental factors on the profiles’ membership values, showing associations between parental factors and youths’ cognition and behavior. The proposed method aligns with the new RDoC^3^ and HiTOP^4^ initiatives and supports the need to broaden the scope of the diagnosis process, as it allows the evaluation of symptomatology in an inclusive diagnostic-agnostic manner. This represents a crucial step towards precision medicine since establishing reproducible and stable profiles across developmental periods will enable the study and understanding of clinical trajectories. To facilitate reaching this goal, we provide all the relevant code to reproduce the results in the form of notebooks (https://github.com/Labo-MAB/Gagnon_FuzzyProfiles_2025) and a Python package allowing researchers to use this framework in new populations (https://github.com/gagnonanthony/NeuroStatX).

## Methods

### Study design and participants

ABCD is a multi-site longitudinal prospective cohort of 11,878 children recruited through school systems across 21 sites in the United States^14^. Children aged 9–11 were enrolled in the study during the 9–11 y follow-up from 2016 to 2018. Recruitment strategies were carefully designed to generate a cohort representing the US sociodemographic population distribution. This is a major advantage compared to other studies, as it enhances the ability to identify and study specific neurodevelopmental trajectories. Of the enrolled participants, 10,843 had completed 9–11 y behavioral, cognitive, and psychopathology data and were included in the present primary analysis. Cognitive and behavioral data from the 11–13 y (*n* = 7,369) and 13–15 y follow-up (*n* = 2846) were also included to evaluate our extracted profiles’ stability across developmental stages. All data were gathered from the data release 5.1 (more details here: https://wiki.abcdstudy.org/). Further details regarding the participating sites, ethics, study protocols, and investigators are available here: https://abcdstudy.org/.

BANDA is a multi-site prospective cohort of 225 adolescents aged 14–17 recruited through clinics, advertisements (social media, buses, and trains), and newsletters^20^. The study is designed to assess brain differences between three clinical groups: depressed, anxious, and control (defined as participants without any diagnosis). Participants were included in the study if they were fluent in English, between 14 and 17 years old, eligible for an MRI, and obtained a score higher than 85 on the Wechsler Abbreviated Scale of Intelligence (WASI). Participants were excluded if they had complications at birth, serious medical conditions, a history of head injury, prior hospitalization for more than 2 days (neurological or cardiovascular disease), a diagnosis of autism spectrum disorder, or used preventive migraine medication daily. Of the enrolled participants, 195 had complete cognitive, behavioral, and psychopathology data from the baseline visit and were included in the present analysis. Further details are available here: https://banda.mit.edu/index.html.

GESTE is a population-based cohort in Sherbrooke, Quebec, Canada^21^. Data used in the present study come from the 9–13 y follow-up in which children aged 9–13 underwent a complete neuropsychological assessment (*n* = 309 participants). Initial enrollment (*n* = 800) happened between 2007 and 2009 during the first trimester or at birth if the participant’s mother met the following criteria: (1) healthy women over 18 years old without severe preterm birth and (2) no chronic medical conditions. Of the participants seen during the fourth follow-up, 271 children with available behavioral, cognitive, and psychopathology data were included in the present analysis. All study protocols were approved by both the Institutional Ethics Boards of the University of Sherbrooke and Columbia University. All population demographics are presented in Table 1.

**Table 1:** Demographics Table for all study populations

	ABCD		BANDA		GESTE	
	Male	Female	Male	Female	Male	Female
**N (%)**	5657 (52.17)	5183 (47.8)	66 (33.85)	129 (66.15)	150 (55.35)	121 (44.65)
**Age, months (std)**	119.1 (7.52)	118.82 (7.49)	181.86 (9.69)	187.18 (9.73)	137.54 (11.83)	136.93 (11.31)
**Race/Ethnicity, count (%)**						
White	3033 (27.97)	2657 (24.5)	49 (25.13)	90 (46.15)	123 (45.39)	98 (36.16)
Black or African American	804 (7.41)	817 (7.53)	1 (0.51)	4 (2.05)	1 (0.37)	-
Hispanic or Latino	1123 (10.36)	1037 (9.56)	5 (2.56)	9 (4.62)	-	2 (0.74)
Asian	110 (1.01)	126 (1.16)	1 (0.51)	6 (3.08)	-	-
Other	587 (5.41)	546 (5.04)	10 (5.13)	20 (10.26)	26 (9.59)	21 (7.75)
**Highest parental education, count (%)**						
No Highschool	238 (2.19)	270 (2.49)	-	-	-	-
Highschool, GED, or equivalent	531 (4.9)	482 (4.45)	-	-	7 (2.58)	6 (2.21)
Some college	1478 (13.63)	1327 (12.24)	9 (4.62)	8 (4.1)	78 (28.78)	57 (21.03)
Bachelor’s Degree	1458 (13.45)	1304 (12.03)	13 (6.67)	37 (18.97)	60 (22.14) ^a^	54 (19.93) ^a^
Postgraduate Degree	1941 (17.9)	1797 (16.57)	44 (22.56)	75 (38.46)		
**Familial Income (USD$), count (%)**						
<50 000$	1053 (9.71)	999 (9.21)	-	-	32 (11.81) ^b^	28 (10.33) ^b^
50 000 - 100 000$	1907 (17.59)	1750 (16.14)	-	-	59 (21.77) ^b^	48 (17.71) ^b^
>100 000$	2211 (20.39)	2002 (18.46)	-	-	37 (13.65) ^b^	26 (9.59) ^b^
**Psychopathology, count (%)**						
AD	563 (5.19)	535 (4.93)	32 (16.41)	36 (18.46)	-	-
ADHD	1103 (10.17)	556 (5.13)	20 (10.26)	24 (12.31)	37 (13.65)	15 (5.54)
CD	212 (1.96)	90 (0.83)	-	-	-	-
DD	23 (0.21)	16 (0.15)	19 (9.74)	61 (31.28)	-	-
OCD	500 (4.61)	320 (2.95)	6 (3.08)	10 (5.13)	-	-
ODD	380 (3.5)	218 (2.01)	5 (2.56)	7 (3.59)	-	-
**Cognitive and Behavioral Scores, mean (std)**^**c**^						
Internalization	−0.00 (0.98)	0.0 (0.98)	−0.03 (0.94)	0.01 (1.02)	0.0 (0.97)	−0.0 (1.01)
Externalization	0.01 (1.06)	−0.0 (0.86)	0.02 (1.13)	−0.0 (0.89)	0.0 (1.0)	−0.0 (0.94)
Stress	0.0 (1.02)	−0.0 (0.92)	−0.0 (1.02)	0.0 (0.97)	0.04 (0.79)	0.03 (0.75)
VA	−0.02 (0.62)	0.03 (0.61)	−0.03 (0.66)	0.02 (0.6)	0.01 (0.66)	−0.02 (0.56)
EFPS	−0.02 (0.49)	0.02 (0.47)	−0.02 (0.5)	0.01 (0.48)	0.01 (0.49)	−0.01 (0.45)
MEM	−0.02 (0.49)	0.02 (0.48)	−0.01 (0.43)	0.01 (0.46)	0.01 (0.5)	−0.01 (0.48)

*GED* General Equivalent Diploma, *AD* Anxiety Disorder, *ADHD* Attention Deficit-Hyperactivity Disorder, *CD* Conduct Disorder, *DD* Depression Disorder, *OCD* Obsessive-Compulsive Disorder, *ODD* Oppositional Defiant Disorder. ^a^Includes all University diplomas (bachelor’s, postgraduates, etc.). ^b^Amounts were converted from CAD to USD currency. ^c^The mean and std values presented were computed after harmonization. Unavailable data or empty groups are marked with a hyphen.

### Procedures

During the 9–11 y follow-up, ABCD participants underwent an exhaustive neurocognitive battery (further described in Luciana et al. ^22^) comprising the NIH Toolbox cognition measures (NIHTB)^23^, the Little Man’s Task (LMT)^24^, the Rey Auditory Verbal Learning Test (RAVLT)^22^, and the Wechsler Intelligence Test for Children-V (WISC-V) Matrix Reasoning task^25^. ABCD participants were readministered the NIHTB^23^ and the LMT^24^ combined with the RAVLT^22^ and the Game of Dice (DICE) task^26^ during the 11–13 y follow-up and the DICE^26^ and the Behavioral Indicator of Resiliency to Distress (BIRD) task^27^ during the 13–15 y follow-up. BANDA participants underwent a similar neurocognitive battery comprising the NIH Toolbox^23^, the University of Pennsylvania Computerized Neuropsychological Test Battery (Penn Test Battery)^28^, and the Wechsler Abbreviated Scale of Intelligence 2nd edition (WASI-II)^29^. For both ABCD and BANDA participants, each test was administered using a computerized version. During the 9–13 y follow-up, at an in-person visit, GESTE participants completed 7 subtests of the Wechsler Intelligence Test for Children-V (WISC-V) test battery^25^. For each study, uncorrected scaled scores were used in further analyses. Following Moore & Conway ^17^, split-sample sequential exploratory factor analysis (EFA) and confirmatory factor analysis (CFA) were performed to obtain latent factors representing three major cognitive domains in each study: verbal ability (VA), executive functions/processing speed (EF/PS), and memory (MEM). Further details regarding the administration, preprocessing, loadings, and fit indices for the EFA/CFA models are available in Supplementary Materials S1–S6, and Tables S1–S10. The mean scaled values of the three latent factors are presented in Table 1.

The parents or caregivers of the ABCD and BANDA participants completed the Child Behavioral Checklist (CBCL) based on their child’s behavior during the six months preceding the follow-up^30^. Using the syndrome and 2007 CBCL scales, scores for internalizing (sum of the anxious/depressed, withdrawn/depressed, and somatic complaints variables), externalizing (sum of the rule-breaking and aggressive behavior variables), and stress problems were computed and included as behavioral indicators. Parents or caregivers of the GESTE participants were asked to complete the Behavior Assessment System for Children 3rd edition (BASC3) parent rating scales. The BASC3 questionnaire is designed to inform on the child’s behavior across four composite scores: externalizing problems (sum of hyperactivity, aggression, and conduct problems subscales), internalizing problems (sum of anxiety, depression, and somatization subscales), behavioral symptoms index (sum of atypicality, withdrawal and attention problems subscales), and adaptive skills (sum of adaptability, social skills, leadership, activities of daily living, and functional communication subscales)^31^. We retained only the externalizing and internalizing composite scores to ease the comparison between studies. Throughout this article, we refer to those variables (externalizing, internalizing, and stress) as “behavioral scores”.

In addition to cognitive and behavioral data, we included available environmental factors encompassing perinatal conditions (maternal age, gestational age, birth weight, substance use, maternal medical conditions, and planned pregnancy), adverse childhood experiences (ACEs; traumatic events, family conflict, and parental psychopathology), sleep hours, neighborhood safety, school factors (environment, disengagement, and involvement), parental factors (acceptance, monitoring, and education level), and economic factors (ability to pay bills, provide food, housing, and medical care) from the ABCD study, as previously conducted to ease comparison with existing results^32^. Available variables from the GESTE study included the perinatal conditions, history of traumatic events, sleep hours, and history of parental psychopathology. Due to the unavailability of such variables in the BANDA study, those participants were excluded from this analysis. Detailed descriptions of variables, instruments, and encoding methods are presented in Supplementary Material S7 and Supplementary Table S11.

A computerized version of the Kiddie Schedule for Affective Disorders and Schizophrenia for School-Aged Children (KSADS) completed by the parent or caregiver was used to assess multiple aspects of the youth’s psychopathology within the ABCD and BANDA study. The KSADS is a widely used tool that highly correlates with DSM-V criteria and has been proven helpful in clinical settings and research studies^33^. Using a similar approach to previous work by Bernanke et al.^33^, we extracted and created categorical diagnosis variables for anxiety disorder (AD), attention deficit-hyperactivity disorder (ADHD), conduct disorder (CD), depressive disorder (DD), obsessive-compulsive disorder (OCD), and oppositional defiant disorder (ODD) (see Supplementary Material S8 for more details). In addition to the parent-administered KSADS, we extracted diagnosis variables from the youth-administered KSADS version from ABCD 9–11 y follow-up. Due to the limited number of modules administered, we only extracted AD and DD categorical diagnosis variables (see Supplementary Material S8 for more details). Diagnosis information from the GESTE participants was pulled directly from the medical records. This article will use the term “psychopathology” to refer to those diagnoses derived from both medical records and questionnaires. Diagnosis distributions are presented in Table 1.

### Statistical analyses

**Fig. 1:**
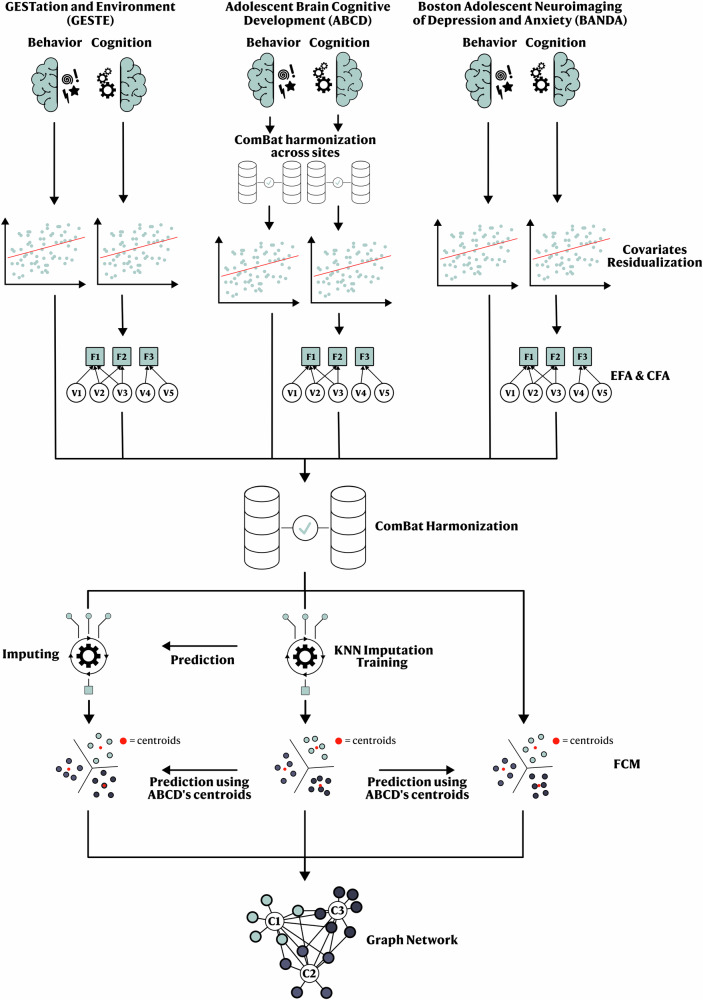
Overview of the statistical pipeline. *EFA* Exploratory Factorial Analysis, *CFA* Confirmatory Factorial Analysis, *KNN* K-nearest Neighbors, *FCM* Fuzzy C-Means.

The Supplementary Materials S2–S6, S9, and S10 provide a complete description of the preprocessing and statistical analyses, while an overview is presented in Fig. 1. Briefly, data from each cohort and follow-up were harmonized (Supplementary Figs. S1–S4) and residualized for covariates (age, sex, ethnicity, and handedness). We imputed the missing Stress variable in the GESTE cohort using a K-Nearest Neighbor model (Supplementary Figs. S5 and S6). The ABCD cohort was used as the discovery sample for all analyses, and both remaining cohorts were used to validate and replicate the results. We applied the fuzzy C-Means (FCM) clustering algorithm to create cognitive and behavioral profiles within the ABCD study as a reference dataset. The optimal number of clusters was derived using the silhouette score (Supplementary Material S10 and Supplementary Fig. S7). Membership values (e.g., the degree to which a participant belongs to a profile, ranging from 0 to 1 for a total sum of 1 across all profiles) were then computed based on the Mahalanobis distance to each profile centroid for each participant (Supplementary Material S10). To enable the comparison between studies, we used the ABCD clusters’ centroids to predict the membership values for each BANDA and GESTE participant. Prediction using the centroids does not require additional studies (used in prediction) to cover all the original extracted profiles or to have a similar sample size. Briefly, using the cognitive and behavioral scores of the “new” participants, the prediction model will “map” the participants by computing the distance from each centroid and then returning the membership values (more details in Supplementary Material S10). A one-way ANOVA followed by a Tukey Honestly Significant Difference (HSD) post hoc test was performed to evaluate the difference in means between profiles and cohorts. When comparing scores or values between profiles, we used the participants’ primary cluster (the highest membership value) as the categorization criteria.

Additional FCM analyses were independently performed (not predicted) for each ABCD follow-up to evaluate the consistency of the extracted profiles across developmental stages using the same preprocessing steps (harmonization and residualization). The extracted profiles were assessed using the same method as the 9-11 y data. To quantify longitudinal stability, we extracted ABCD participants with available data for all three follow-ups (*n* = 2359). We computed the percentage of participants who remained in the same main profile (defined as the profile with higher membership value) between the 9–11 year, 11–13 year, and 13–15 year follow-ups as an index of stability over time. Participants’ movement across time points was evaluated using a movement ratio, calculated by dividing the number of participants moving out of their main profile by the total number of participants in the original profile.

Clusters were visualized by constructing a weighted graph network using the participants as nodes and membership values as the edge’s weight^34^. To quantify the non-random distribution of psychopathology on the graph network, we computed the average shortest weighted path (ASWP) between all nodes of interest. A higher value translates to a more compact aggregation and a non-random distribution. Permutation testing using 5000 iterations was performed to estimate the results’ significance. We conducted two partial least squares regression analyses (PLSR) using environmental factors as predictors and the profiles’ membership values as dependent variables in ABCD and GESTE only. BANDA participants were excluded from this analysis since no environmental factors were collected during the study. Membership values for each profile were included as continuous variables, leveraging the ability of PLSR to handle multicollinearity. PLSR extracts components for predictors and dependent variables to maximize the covariance between those two sets and returns coefficients for each predictor to each dependent variable^35^. Those coefficients can be used to understand the influence of the predictors on the dependent variables. For example, while it does not reflect a direct linear relationship due to the projection into latent space in PLSR, a high coefficient for a predictor will reflect a higher dependent variable if that predictor’s value increases. In other words, the sign of the coefficient reflects its directionality, while its magnitude reflects relative importance^35^. Ten-fold cross-validation was used to assess the optimal number of components. Since cohorts do not have corresponding environmental variables, independent models were fitted, resulting in distinct components for each cohort. Permutation testing using 10,000 iterations assessed the model and coefficients’ significance. All reported p-values are corrected for false discovery rate (FDR), and the post-correction significance threshold was set to *p*_fdr_ < 0.05^36^. Complete code to reproduce the analyses is available here: https://github.com/Labo-MAB/Gagnon_FuzzyProfiles_2025. A Python package containing general-use command-line scripts allowing researchers to use this method in new populations is available here: https://github.com/gagnonanthony/NeuroStatX.

## Results

From the participants with complete data, 10,843 ABCD participants for the 9–11 y follow-up were included; meanwhile, 195 and 271 participants were retained from the BANDA and GESTE studies, respectively. Demographic information is presented in Table 1. To evaluate the stability of the profiles across developmental stages, 7369 and 2846 participants from ABCD’s 11–13 y and 13–15 y follow-ups were included (Supplementary Table S12).

### Cognitive and behavioral fuzzy profiles

For each cohort, we extracted three latent cognitive factors (verbal ability, executive function/processing speed, and memory) using split-sample exploratory and confirmatory factor analysis as proposed in previous studies^16,17^. Additionally, we extracted three behavioral scores (internalization, externalization, and stress) from validated questionnaires. Then, cognitive factors and behavioral scores were residualized for covariates and included in the fuzzy clustering analysis (see Methods, Fig. 1, and Table 1).

**Fig. 2:**
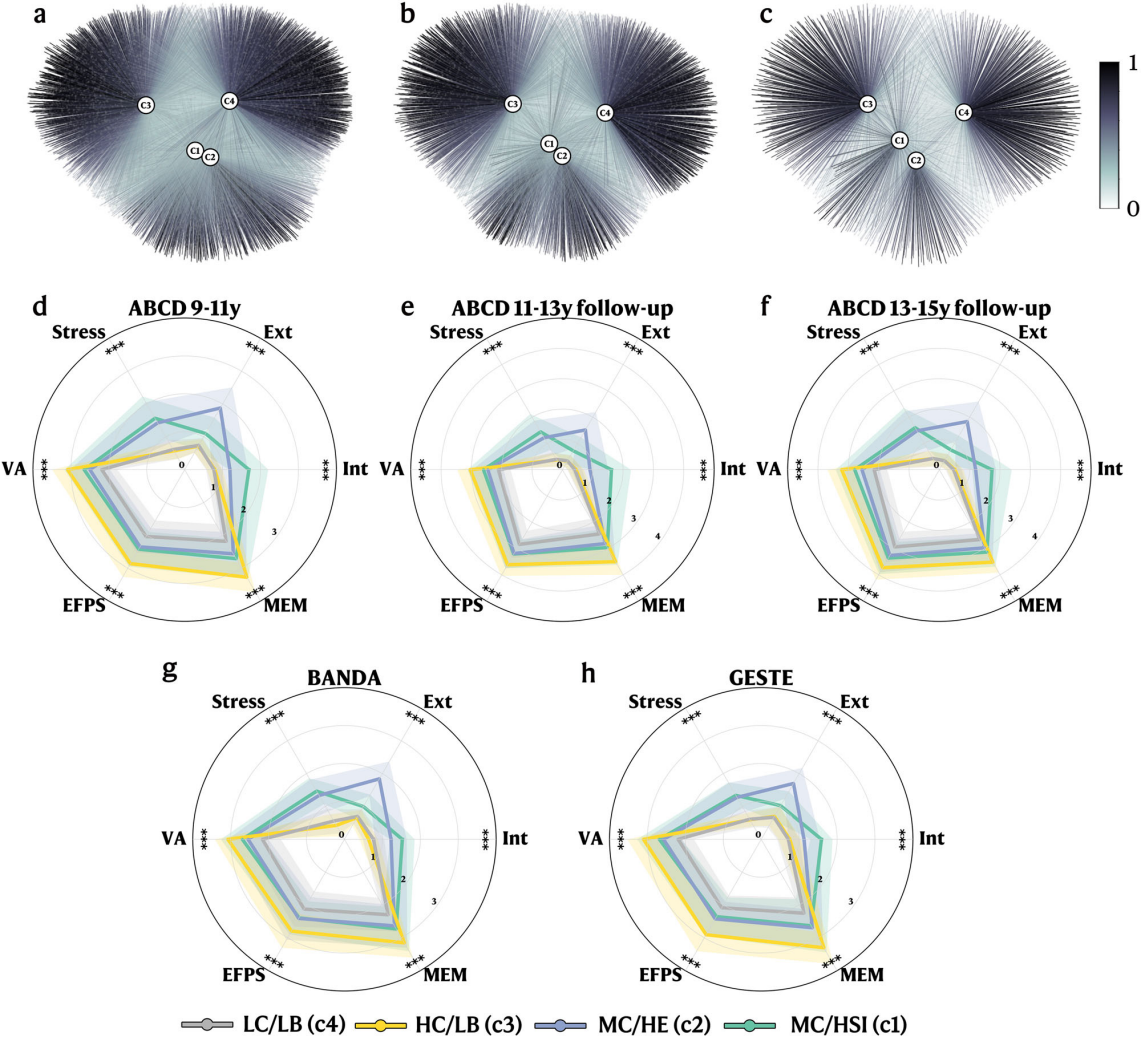
FCM cognitive and behavioral profiles. **a** Graph network representing the FCM clustering results (on the ABCD 9-11 y data), including the mapped BANDA and GESTE datasets. Grey nodes represent subjects, and edges represent the membership values between a single subject and each cluster’s centroid. **b**, **c** Graph networks representing the independent FCM clustering results for ABCD 11-13 y and 13–15 y follow-ups. **d**–**h** Radar plot of the mean values (with standard deviation) stratified by clusters for each behavioral symptom and cognitive variable for each cohort or follow-up. **d** ABCD 9-11 y follow-up. **e** ABCD 11–13 y follow-up. **f** ABCD 13–15 y follow-up. **g**. BANDA. **h** GESTE. Participants were grouped based on their primary profile (the highest membership value) for the radar plots. ***p < 0.001. Scores are scaled for visualization purposes. VA Verbal Ability, EFPS Executive Function and Processing Speed, MEM Memory, Ext Externalization, Int Internalization, MC/HSI Profile with moderate cognitive scores and high stress/internalizing behavioral symptoms, MC/HE Profile with moderate cognitive scores and high externalizing behavioral symptoms, HC/LB Profile with high cognitive scores and low behavioral symptoms, LC/LB Profile with low cognitive scores and low behavioral symptoms

The 9–11 y ABCD follow-up returned an optimal 4-cluster solution, derived using the silhouette score, representing four different behavioral and cognitive profiles (Fig. 2, Supplementary Fig. S7). Using the participants’ highest membership value as their primary profile, profiles C3 and C4 contain most study participants (*n* = 4032 and 3730, respectively) and represent low behavioral scores with high (HC/LB) and low (LC/LB) cognitive scores, respectively (Fig. 2). Profiles C1 and C2 contain fewer study participants (*n* = 1896 and 1651, respectively) and show moderate cognitive scores with high stress/internalizing behavior (MC/HSI) and high externalizing behavior scores (MC/HE), respectively (Fig. 2). Interestingly, participants with higher behavior scores (all domains included) were only associated with moderate cognitive capabilities, not low or high cognitive capabilities.

### Longitudinal stability of the profiles

**Fig. 3:**
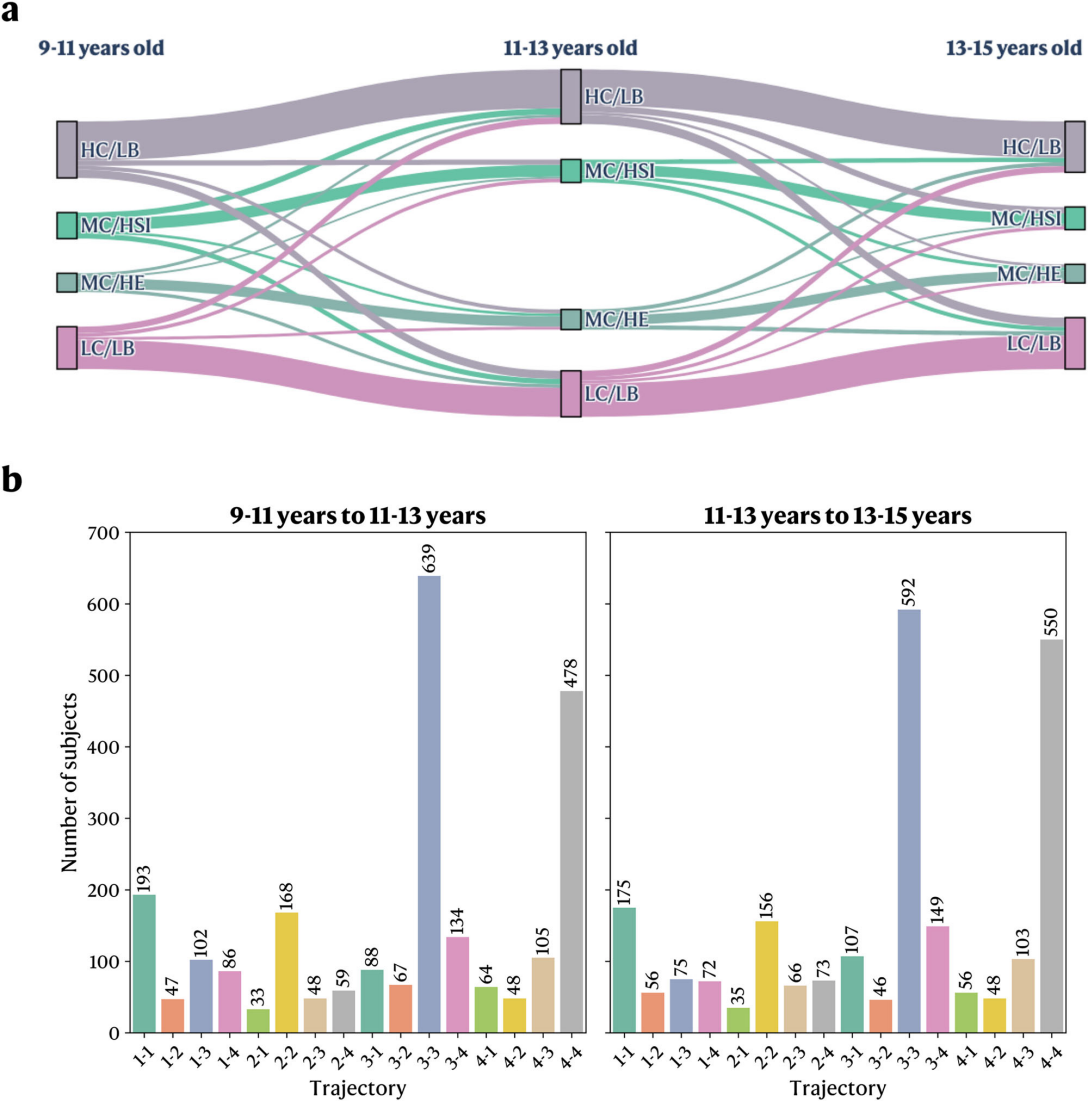
Sankey diagram of the longitudinal movement across profiles between the 9-11 years, 11-13 years, and 13-15 years follow-ups. **a** Sankey diagram of the movement across profiles for the three time points. The size of the band represents the number of participants in this trajectory. **b** Bar plots representing the amount of participants that followed a specific trajectory. Highest membership value was used to determine the main profile. Each pair of numbers on the x-axis represents the initial and the final profile, respectively. MC/HSI (1): Profile with moderate cognitive scores and high stress/internalizing behavioral symptoms. MC/HE (2): Profile with moderate cognitive scores and high externalizing behavioral symptoms. HC/LB (3): Profile with high cognitive scores and low behavioral symptoms. LC/LB (4): Profile with low cognitive scores and low behavioral symptoms.

To evaluate the stability across developmental stages of the extracted profiles, we performed additional independent FCM analysis on the 11–13 y and 13–15 y follow-ups using identical methods and input variables as 9–11 y follow-up (see Methods). Both returned nearly identical profiles, with profiles C3 (HC/LB) and C4 (LC/LB) containing most participants (11–13 y: *n* = 2631 and 2504, 13–15 y: *n* = 1015 and 1018, respectively) while profiles C1 (MC/HSI) and C2 (MC/HE) contain fewer participants (11–13 y: *n* = 1130 and 1104, 13–15 y: 436 and 377) (Fig. 2). Participants exhibiting higher behavioral scores on all scales were again associated with moderate cognitive abilities. To quantify the stability across time of the profiles, we evaluated the percentage of participants who did not move profiles across timepoints, revealing that 62.65% and 62.44% of participants remained in the same profile between the 9–11 years to 11–13 years and 11–13 years to 13–15 years follow-ups, respectively (Fig. 3a). Furthermore, we calculated a movement ratio, which highlights the ratio of participants moving out of a profile between two time points compared to the initial number of participants within that profile. Results showed that profiles MC/HE and MC/HSI showed between 14 and 27% more movement across both time points than profiles HC/LB and LC/LB (Table 2). When examining the most common next destination for participants moving out of their initial profile, we found that participants transitioning out of profiles MC/HSI or MC/HE were most likely to land in profiles HC/LB or LC/LB at both time intervals (Fig. 3b). Most participants moving out of profile LC/LB were found in profile HC/LB at the subsequent follow-up (for both time intervals); the opposite was found for participants moving out of profile HC/LB (Fig. 3b).

**Table 2:** Movement ratio in the ABCD cohort between the 9–11 years, 11–13 years, and 13–15 years follow-ups

	9–11 years to 11–13 years	11–13 years to 13–15 years
**Movement ratio (%)**		
MC/HSI	0.55 (54.91%)	0.54 (53.70%)
MC/HE	0.45 (45.45%)	0.53 (52.73%)
HC/LB	0.31 (31.14%)	0.34 (33.78%)
LC/LB	0.31 (31.22%)	0.27 (27.34%)

The ratio is defined as the number of participants moving out of the profiles divided by the number of participants initially within this profile. MC/HSI: Profile with moderate cognitive scores and high stress/internalizing behavioral symptoms. MC/HE: Profile with moderate cognitive scores and high externalizing behavioral symptoms. HC/LB: Profile with high cognitive scores and low behavioral symptoms. LC/LB: Profile with low cognitive scores and low behavioral symptoms.

### Generalizability to external populations

Generalizable results are key to reproducible and sustainable science; therefore, we assess the replicability and robustness of our profiles in two external cohorts. After harmonization, we predicted the membership values for the BANDA and GESTE participants using ABCD clusters’ centroids (see Methods). The prediction process relies on mapping the participants onto the existing ABCD profiles and computing their distance from each centroid. Prediction does not need complete coverage of all profiles and is suitable for studies of smaller sizes (see Methods). Both BANDA profiles (HC/LB: 59, LC/LB: 55, MC/HSI: 47, and MC/HE: 34 participants) and GESTE profiles (HC/LB: 83, LC/LB: 77, MC/HSI: 58, and MC/HE: 53 participants) showed close-to-identical score patterns, highlighting their replicability and robustness across various populations (Fig. 2 and Supplementary Fig. S8). The difference in means between each profile for each cohort and between cohorts is provided in Supplementary Tables S13–S18.

### Consistency with clinical diagnoses

**Fig. 4:**
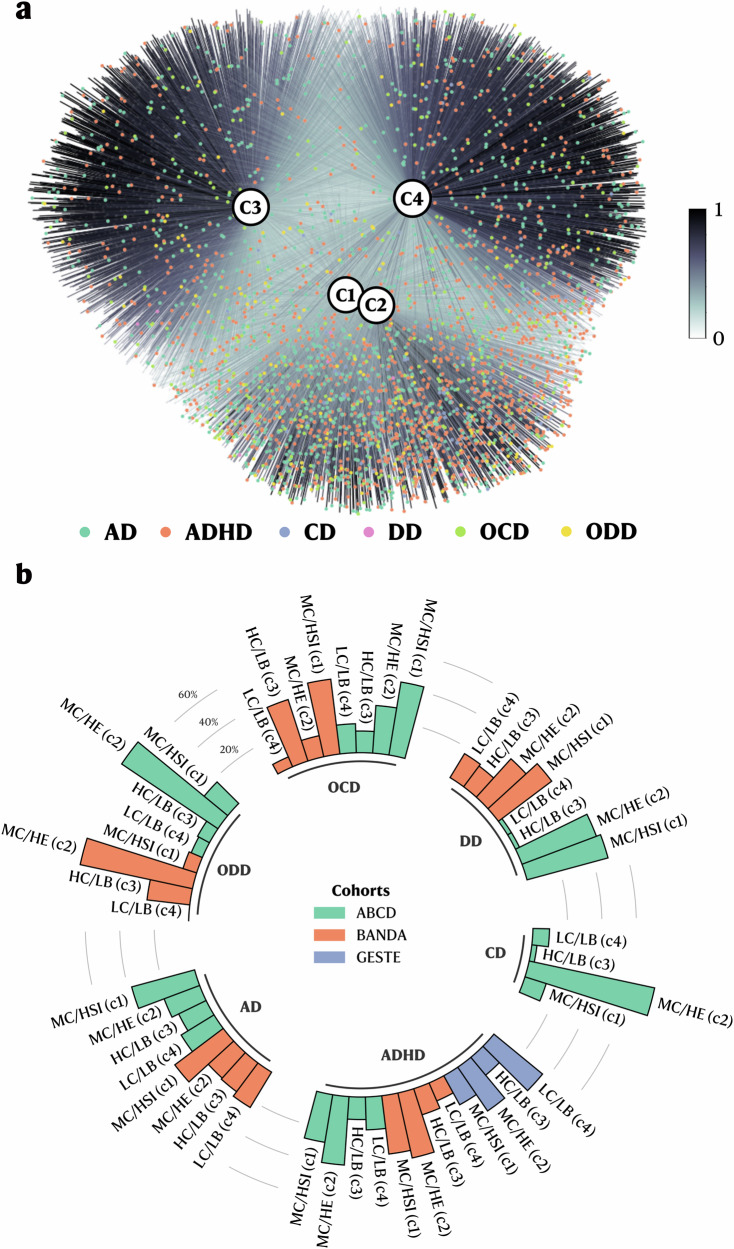
Diagnosis distribution across all profiles. **a** Graph network including all cohorts (ABCD 9-11 y, BANDA, and GESTE) with participants colored based on their psychiatric disorder (assessed either via parental K-SADS or from experts). Darker edges reflect higher membership values. **b** Circular bar plot showing the percentage of participants with a diagnosis across all the extracted clusters and cohorts. Participants were grouped based on their primary profile (the highest membership value) for the bar plot. AD Anxiety Disorder, ADHD Attention Deficit-Hyperactivity Disorder, CD Conduct Disorder, DD Depression Disorder, OCD Obsessive-Compulsive Disorder, ODD Oppositional Defiant Disorder, MC/HSI (1) Profile with moderate cognitive scores and high stress/internalizing behavioral symptoms, MC/HE (2) Profile with moderate cognitive scores and high externalizing behavioral symptoms, HC/LB (3) Profile with high cognitive scores and low behavioral symptoms, LC/LB (4) Profile with low cognitive scores and low behavioral symptoms.

Extracting profiles from populational data is relatively easy. However, extracting *meaningful* profiles from populational data is a more challenging task. To validate that the proposed profiles had clinical utility, we evaluated their consistency with existing DSM-V diagnoses. While diagnoses have limitations surrounding heterogeneity and overlap between clinical populations, they represent the current clinical standard that should be reflected within the profiles with expected differences. First, we projected the profiles into a graph network, labeling nodes as participants and edges as membership values; then, we mapped participants with a diagnosis and evaluated their distribution pattern (Fig. 4 and Supplementary Fig. S9). Considering the highest membership value as the participants’ main profile, we assessed the distribution of anxiety disorder (AD), attention deficit-hyperactivity disorder (ADHD), conduct disorder (CD), depressive disorder (DD), obsessive-compulsive disorder (OCD), and oppositional defiant disorder (ODD) obtained from the parent-administered Kiddie Schedule for Affective Disorders and Schizophrenia for School-Aged Children (KSADS) (ABCD and BANDA) and the medical records (GESTE) (see Methods and Table 1). We found that participants with disorders characterized by externalizing behaviors (ADHD, CD, and ODD) were mainly within the MC/HE profile in the ABCD (39.82%, 74.50%, and 67.39%, respectively) and BANDA studies (ADHD: 38.64%, and ODD: 66.67%) (Fig. 4). However, in the GESTE study, in which psychopathology was pulled from medical records, ADHD participants were split between the LC/LB and MC/HE profiles (36.54% and 30.77%, respectively) (Fig. 4). Participants with AD or OCD were marginally more present in profile MC/HSI in the ABCD study (37.85% and 42.93%, respectively) (Fig. 4). In contrast, participants with a DD diagnosis were equally found within profile MC/HSI and MC/HE in the ABCD and BANDA studies (ABCD: 48.72% and 46.15%, respectively, and BANDA: 38.75% and 30.00%, respectively) (Fig. 4). However, AD and OCD in the BANDA study were also found in the MC/HSI profile (35.20% and 43.75%, respectively), whereas OCD was also prevalent in the HC/LB profile (37.50%) (Fig. 4). Using the youth-administered KSADS in the ABCD cohort, AD participants were mainly found within the MC/HSI profile (39.06%). In contrast, DD participants were found within the LC/LB, MC/HSI, and MC/HE profiles (36.14%, 25.30%, and 24.10%, respectively) (Supplementary Fig. S10).

We leveraged graph theory concepts to compute the average weighted shortest path (ASWP) between participants with a specific diagnosis (see Methods). We compared this to a null distribution (e.g., an equally randomly selected number of participants), where significant p-values would reflect a non-random distribution of participants (e.g., they tend to aggregate in specific profiles) (see Methods). Additionally, we created a PSYPATHO index, representing participants who had at least one psychiatric disorder, to examine overall concordance with psychopathology. Overall, each study and assessment method diagnosis met the FDR-corrected significance threshold for a non-random distribution (Table 3).

**Table 3:** Average shortest weighted path (ASWP) for all diagnoses in the ABCD 9-11 y follow-up, BANDA, and GESTE populations

	ABCD (parent KSADS)		ABCD (youth KSADS)		BANDA		GESTE	
	ASWP	*p*_fdr_	ASWP	*p*_fdr_	ASWP	*p*_fdr_	ASWP	*p*_fdr_
**AD**	0.214	0.0002	0.217	0.0003	0.194	0.012	−	−
**ADHD**	0.225	0.0002	−	−	0.226	0.001	0.206	0.012
**CD**	0.276	0.0002	-	-	-	-	-	-
**DD**	0.294	0.0002	0.208	0.0004	0.212	0.001	-	-
**OCD**	0.234	0.0002	-	-	0.223	0.009	-	-
**ODD**	0.260	0.0002	-	-	0.220	0.012	-	-
**PSYPATHO**	0.214	0.0002	0.213	0.0003	0.197	0.009	0.206	0.012

*AD Anxiety Disorder, ADHD* Attention Deficit-Hyperactivity Disorder, *CD* Conduct Disorder, *DD* Depression Disorder, *OCD* Obsessive-Compulsive Disorder, *ODD* Oppositional Defiant Disorder, *PSYPATHO* Index indicating participants with at least one psychiatric disorder (a value of 1 means that the participant had at least one psychiatric disorder), *p*_fdr_ FDR-corrected p-value.

### Solving challenging real-world questions: impact of environmental factors

**Fig. 5:**
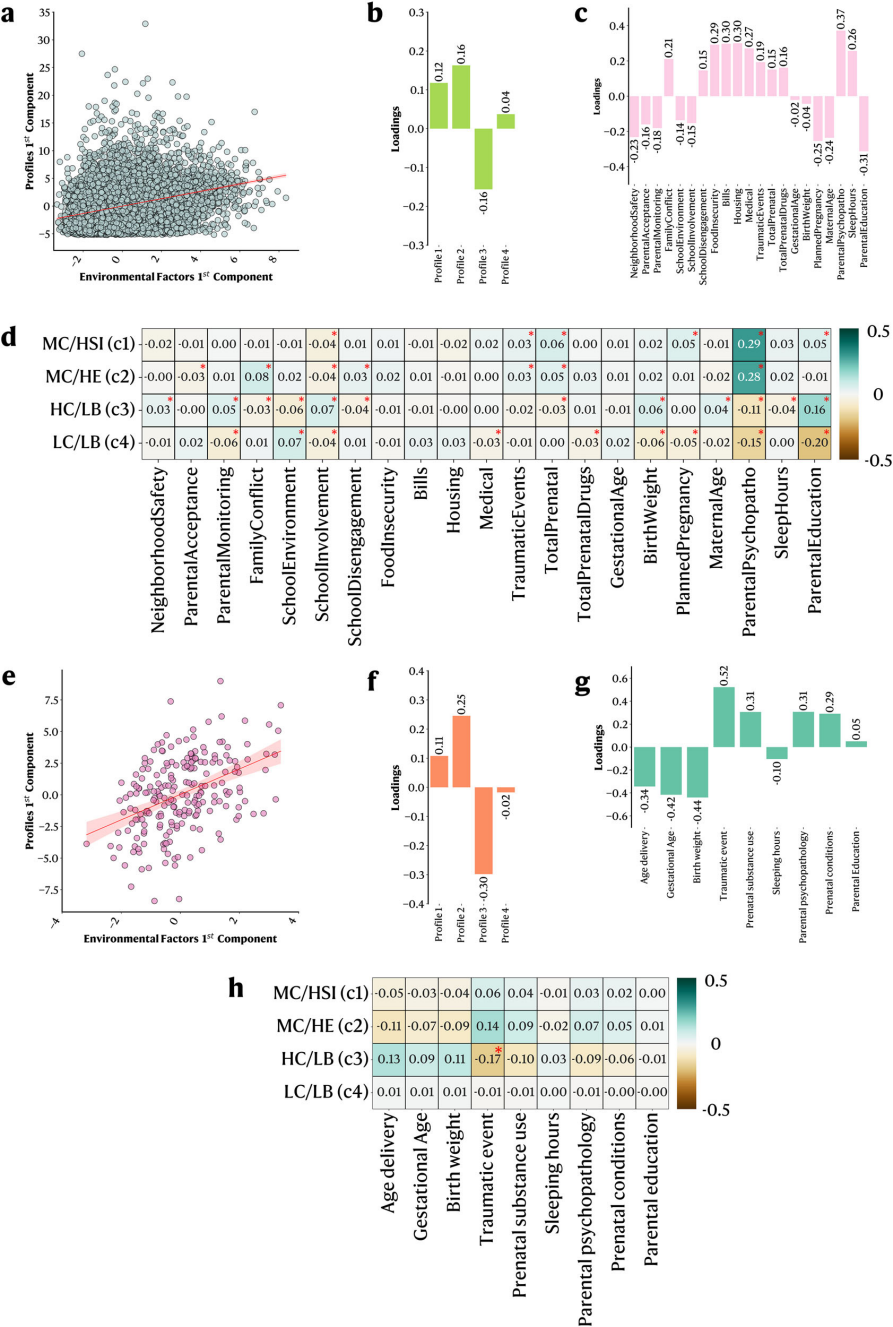
Partial Least Squares Regression (PLSR) results between environmental factors and cognitive/behavioral profiles in the ABCD and GESTE cohort. PLSR analysis between environmental factors and cognitive/behavioral profiles in the ABCD 9-11 y (**a**–**d**) and GESTE (**e**–**h**) studies. Scatter plots of the 1st profile and 1st environmental components in ABCD (**a**) and GESTE (**e**). Those represent the first extracted pair of components that maximize the variance between the environmental factors and the profile membership values in the PLSR models. Loadings on each first component (**b**, **f** profile’s component, and **c**, **g** environmental components). Higher values (either positive or negative) reflect a higher contribution to the component. Heatmaps of the variables’ coefficients for each profile in ABCD (**d**) and GESTE (**h**). MC/HSI (1): Profile with moderate cognitive scores and high stress/internalizing behavioral symptoms. MC/HE (2): Profile with moderate cognitive scores and high externalizing behavioral symptoms. *HC/LB (3)* Profile with high cognitive scores and low behavioral symptoms, *LC/LB* (4) Profile with low cognitive scores and low behavioral symptoms. *: p_fdr_ < 0.05.

The cognitive and behavioral profiles proposed here extract meaningful patterns from the data while retaining all available information from individual participants’ cognitive abilities and behavioral manifestations. To showcase their potential in investigating relationships with external factors, we evaluated the relationship between environmental factors and the cognitive/behavioral profiles in the ABCD and GESTE cohorts. Participants from the BANDA study did not have data on environmental factors and were therefore excluded from the analysis. One key aspect to consider is the collinearity between each profile membership value. Consequently, we performed two partial least squares regression analyses (PLSR, one for each cohort) to handle collinearity, and we established the significance of both models and coefficients using permutation testing. We present the first extracted components (environmental component and profile component) and the variable loadings for both cohorts in Fig. 5. Higher loading values (either positive or negative) reflect a higher contribution to the extracted component. PLSR returns coefficients that can be further used to understand the directionality and magnitude of the association between environmental factors and the membership values of cognitive and behavioral profiles (see Methods)^35^.

PLSR models were significant in both ABCD (R^2^ = 0.10, *p* < 0.001) and GESTE (R^2^ = 0.06, *p* = 0.002) cohorts. In the ABCD study, participants showing lower school involvement, higher traumatic events, higher prenatal conditions (total conditions and planned pregnancy), higher parental psychopathology, and higher parental education had higher membership value in the MC/HSI profile (Fig. 5). Similar trends for school involvement, traumatic events, total prenatal conditions, and parental psychopathology were also predictive of participants in the MC/HE profile, in addition to lower parental acceptance, higher family conflict, and higher school disengagement (Fig. 5). Participants’ membership value to profile HC/LB was mainly influenced by higher neighborhood safety, higher parental monitoring, lower family conflict, lower school environment, higher school involvement, lower school disengagement, lower total prenatal conditions, higher birthweight, higher maternal age, lower parental psychopathology, lower sleep hours, and higher parental education (Fig. 5). Opposingly, lower parental monitoring, higher school environment, lower school involvement, lower birthweight, and lower parental education were driving higher membership values in profile LC/LB combined with a lower ability to pay medical bills, lower prenatal exposure to drugs, less planned pregnancy, and lower parental psychopathology (Fig. 5). In the GESTE study, similar trends were observed. Still, only lower experiences of traumatic events were predictive of participants in profile HC/LB and reached the significance threshold after FDR correction (Fig. 5).

## Discussion

Although some studies have attempted to extract profiles from populational data, none succeeded in embracing the known continuous nature of cognition and behavior found in the general population. Using data-driven fuzzy clustering, we successfully extracted a 4-profile solution representing the continuum of cognition and behavior in a prominent pediatric cohort. Using this referential model, we demonstrated the generalizability of the cognitive and behavioral profiles in two additional independent pediatric cohorts, enabling the direct comparison between studies. Furthermore, we independently reproduced those profiles in subsequent follow-ups within the ABCD study, showcasing their stability across developmental periods.

Previous population stratification studies found that the majority of their participants were in the low-symptom groups^18^. We report similar results; both low behavioral symptom profiles accounted for the largest number of participants. Studies that included both internalizing and externalizing symptoms reported stratified subgroups of internalizing or externalizing behavior^37,^^38^, which is consistent with our current results. Existing studies have also leveraged profiling/clustering techniques to stratify cognitive abilities, reporting a high-moderate-low abilities stratification in multiple contexts^39,40^, similar to our current results. Although our multidimensional method encompasses both cognition and behavior, our profiles align with previous subgroup stratification studies, both in cognition and behavior, indicating that they capture meaningful subgroups that could be useful in clinical contexts. While we observed strong replication of the 4-profile solution across all cohorts and follow-ups, the profiles MC/HE and MC/HSI in the BANDA and GESTE cohorts did not show a statistically significant difference in cognitive scores (Supplementary Tables S14 and S15). While those comparisons are statistically significant in ABCD, they may be a product of the high sample size, where minor differences between groups can more easily become significant due to increased statistical power, currently lacking in BANDA and GESTE. More importantly, both profiles remained significantly different from the high- and low-cognitive profiles (HC/LB and LC/LB), reinforcing their unique behavioral characteristics. From a clinical perspective, those findings highlight the possibility of divergent behavioral symptoms (externalizing vs. internalizing) even in the absence of meaningful cognitive differentiation. In transdiagnostic frameworks such as RDoC^3^ or HiTOP^4^, this stratification is essential, as it supports the notion that within a specific dimension or construct, one can exhibit distinct behavioral symptoms.

Additionally, in all included cohorts and follow-ups, children with moderate cognitive abilities tend to show more behavioral manifestations (externalizing, internalizing, or stress) than children with high or low cognitive abilities. This suggests that the relationship between cognition and behavior might not be linear, significantly contrasting with previously published results^16,17,19,41–43^. However, a recent study questioned that linearity and found that non-linear models were better suited to explain the relationship between cognition and behavior^44^, further supporting our analysis. This contradiction between results highlights the sensitivity of our method since it uncovered the non-linear relationship between cognition and behavior, which other studies could not. It also highlights the advantage of our method compared to purely dimensional approaches. Our profiles can derive higher-order populational structure representing patterns of co-occurring traits across individuals, such as the non-linear relationship between cognition and behavior. While it provides information at the population level, it also offers precise individual-level information, successfully acting as a bridge between dimensional and categorical approaches. In the clinical context, clinicians would have access to both interpretable profiles, allowing for risk assessment and monitoring, as well as detailed information on symptom overlap at the patient level. As such, our method provides an interpretable yet flexible framework that mirrors clinical reality, where symptom overlap and diagnostic ambiguity are common^2^.

When evaluating the temporal stability of the profiles at the individual level, our results showed that nearly two-thirds of all participants remained in the same profile across time points (either between 9-11 years and 11-13 years, or 11-13 years and 13-15 years). Those results indicate moderate stability across developmental stages, not only at the population level, but also at the individual level. Additionally, further analyses revealed that profiles exhibiting higher behavioral symptoms showed greater movement across time points, indicating greater volatility in symptom presentation throughout childhood and adolescence. Those results align with previous literature suggesting that internalizing and externalizing symptoms exhibit small to moderate stability across this developmental period^45^. Such instability could also stem from the ongoing neurodevelopmental reorganization, maturation of self-regulatory processes, and rapidly changing environmental and social contexts in early adolescence^11,12,46^. In the clinical context, these observed transitions could also reflect successful interventions or the emergence of compensatory mechanisms. This ability to derive trajectories for a single participant could be beneficial for clinicians, as it would allow them to monitor the effect of interventions, environmental, and/or social changes.

Consistency with existing clinical diagnoses is a key validation step for studies aiming to extract, using data-driven methods, subgroups or profiles from the general population. The current analysis did not aim to predict diagnostic labels, but rather serves as an initial validation step to support the clinical relevance of the profiles. While it is expected that some differences emerge, considering the known heterogeneity in psychopathology^1,2^, clinical diagnoses remain the gold standard benchmark. Our method was consistent with the existing clinical domains, noting only differences between parent-reported, youth-reported, or clinician-derived diagnoses. Differences between youth-reported and parent-reported diagnoses are known^47^, especially concerning internalizing behavior^48^. Parents often exacerbate their child’s symptoms, whereas youths understate them^48^. However, differences between parent-reported and expert diagnostic opinions are puzzling and suggest substantial implications for research settings, where parent-reported diagnoses are often used as group classifiers^33^. Compared to clinical diagnosis, parental assessment is likely affected by multiple bias sources based on their cultural background and socioeconomic situation. Additionally, participants with a clinical diagnosis were most likely under medication and/or following some behavioral interventions. Since behavioral assessment is reported based on the 6 months preceding the visit to the research center, it may reflect the success of the intervention in those children, resulting in lower behavioral scores across dimensions. While the current study lacks data and sample size in the expert diagnostic opinions category for a concrete conclusion, it highlights the need for a diagnostic-agnostic approach as proposed here. Our method alleviates this issue by modeling cognition and behavior as non-discrete profiles.

One major current goal in psychiatry is the transition toward precision medicine, which involves including biological and/or environmental factors combined with new data-driven machine-learning methods in the diagnosis process^5,49,50^. To illustrate a case study using our method, we investigated the relationship between our extracted cognitive and behavioral profiles and environmental factors in two of the included cohorts. Our results suggest a strong implication of parental education, lower prenatal substance use, and lower parental psychopathology in cognitive abilities, consistent with previously reported results^15,32,51^. It further supports the hypothesis that higher-education parents are more likely to invest time (e.g., reading books) and resources in their child’s cognitive development^52^ and might even reinforce the genetic contribution behind cognitive abilities. Unsurprisingly, similar to previous studies, parental psychopathology was highly implicated in profiles showing high behavioral scores (externalizing and internalizing/stress) in addition to prenatal conditions, family conflict, and the experience of traumatic events^15,51^. Indeed, parental behavioral problems are highly predictive of offspring’s psychopathology^53^ and, combined with other environmental factors, are thought to influence the brain’s developmental trajectories^53–57^. Part of this relationship can be explained by the heritability of psychopathology, as shown in genetic studies^58^. Although it is hard to separate the genetic contribution from the environmental factors in the current data, common genetic variants have been shown to have transdiagnostic influences^58^, suggesting fuzzy boundaries even at the genetic level and not only in cognitive/behavioral phenotypes. This demonstrates that our method can derive meaningful relationships between external factors (e.g., environmental factors), cognition, and behavior while retaining all the available information compared to discrete classification methods. Future studies could reuse our method to investigate the underlying neural correlates, as previous studies have reported associations between brain structures and environmental factors^53–57,59,60^. Specifically, future studies should investigate the relationship between neurophysiological processes, the movement between profiles across time in participants, and how those movements are triggered during adolescents’ development.

Although our method is data-driven, the selection of our behavioral and cognitive constructs to include in our profiling approach was guided by theoretical and clinical foundations. Indeed, externalizing behaviors in childhood have been prospectively linked to antisocial behavior, substance use, and affective disorder in adulthood^9,61^. Similarly, impairments in executive functioning, verbal ability, and memory are well-documented early markers in individuals at clinical high risk for psychosis and in youth with ADHD^62,63^. Internalizing and stress symptoms in preadolescents, including anxiety and mood dysregulation, have been considered significant predictors of later depression^64^. Moreover, the included cognitive and behavioral constructs were assessed using widely adopted instruments across both research and clinical settings, enhancing the translational value of our method. The longitudinal and out-of-sample stability of the profiles further supports their robustness, suggesting that they capture stable, transdiagnostic, and meaningful patterns of individual differences relevant to psychiatric risk.

Furthermore, our method is ideally suited for a precision medicine framework as it is generalizable and can be used on a single individual. One crucial advantage of data-driven fuzzy clustering over other methods is the ability to quantify the level of belonging to each profile for each subject. While other methods, such as latent profile analysis, can consider classification uncertainty using probabilities, there are fundamental differences with the proposed approach. Probabilities refer to the likelihood that an event occurs, which, in the case of classification, is the likelihood that a participant is part of a profile. It does not indicate to what degree an individual is part of that profile, but quantifies how confident we are with the discrete classification. Fuzzy logic captures the meaning of partial truth, where an individual can simultaneously belong to more than one profile to various degrees (membership values). Those fundamental differences make fuzzy clustering better suited when there is known overlap and when individuals can exhibit characteristics from multiple profiles. It is known that cognition and behavior represent an overlapping continuum of capabilities/manifestations; therefore, embracing the fuzziness in a general population is mandatory and most likely better reflects the real-world scenario compared to subgroup extraction or probabilistic methods.

Our method also provides a novel perspective on understanding the developmental origins of psychiatric vulnerability. We sought to characterize early, multidimensional phenotypes that may shape individual trajectories over time. This approach reflects a shift from categorical diagnosis toward developmental risk modeling, based on the growing knowledge that psychopathology in young adulthood is often preceded by complex, qualitatively distinct patterns in late childhood^11,46^. In the clinic, our profiles could be interpreted as data-driven analogues to clinical prodromes, capturing heterogeneity that clinicians may already recognize but cannot easily quantify. For instance, a child with moderate cognitive functions and elevated internalizing symptoms may not meet criteria for a specific disorder yet could be on a pathway toward affective or anxiety-related difficulties in adolescence and early adulthood. The included behavioral and cognitive constructs are already vastly embedded in multidisciplinary assessments (e.g., pediatric neuropsychology, child psychiatry). The integration of these domains into a single profiling framework can facilitate early characterization of risk, support the tailoring of intervention strategies, and guide monitoring across development. The long-term goal would be to support predictive modeling of psychiatric trajectories based on our profiles, similar to the use of the Framingham equation for cardiovascular risk^65^ or the frailty biomarkers in aging populations^66^. This would provide a probabilistic tool to identify vulnerable individuals early, while their brain networks are still malleable^10,11^. This would also open the door for scalable implementation in community and primary care settings, particularly in the context of transdiagnostic screening.

However, our study also has important limitations to consider. First, not all data collection instruments were identical across studies, and cohort selection was based on the availability of these detailed phenotyping instruments and corresponding data, which could have introduced an opportunistic selection bias into our method and limited its applicability to populations where detailed phenotyping is not available. While this could limit our reproducibility evaluation in different populations, we mitigated this risk using robust harmonization techniques (see Supplementary Fig. S4). Second, the stress variable was unavailable in the GESTE cohort; thus, we imputed it using a sophisticated imputation model and assessed its performance on existing data and against an independent variable closely related to the imputed one (see Supplementary Figs. S5 & S6). This imputed variable might have introduced an overestimation of the membership values for profile MC/HSI in the GESTE cohort. Third, we evaluated the relationship with the available environmental factors within our cohorts. However, those factors only capture a subset of the possible environmental exposures. Future studies should include additional variables, such as exposure to substances or diet-related factors, to the ones used in this study. Fourth, all included cohorts were from North American backgrounds (Canada and the USA), which might introduce cultural bias in our results. Future studies should validate the results in other cultural backgrounds.

While this study only examines individual-level relationships, our framework could also be applied to examine population-level relationships, such as the impact of famine and war, which would shift the current profiles’ distribution. This aspect highlights the granularity of the method and the wide range of possible applications. One critical step to attaining precision medicine is the ability to create generalizable frameworks for various populations. Hence, we validated our fuzzy profiles in two smaller independent cohorts (GESTE and BANDA) and across developmental stages using subsequent ABCD follow-ups, demonstrating the generalizability and replicability of the profiles. Additionally, we ensured consistency with existing clinical domains, showing good-to-great concordance and reinforcing the usability of our method in clinical settings. Finally, we showcased how our method could be used to investigate pressing research questions by looking at the relationship between the profiles and environmental factors. Establishing this referential in the ABCD cohort expands the applicability to other smaller cohorts, where statistical power might be insufficient to extract similar profiles.

## Supplementary information

Supplementary Material

## Data Availability

The Adolescent Brain and Cognitive Development (ABCD) and the Boston Adolescent Neuroimaging of Depression and Anxiety (BANDA) are freely available datasets accessible via the NIMH Data Archive platform (https://nda.nih.gov/). Researchers must apply for a Data Use Certificate, which will be reviewed and granted by study administrators. Data for the GESTation and Environment (GESTE) cohort is not publicly available but can be accessed by contacting the principal investigator in charge of the study, Dr. Larissa Takser (larissa.takser@usherbrooke.ca). Code to reproduce the findings, including data curation, preprocessing, analysis, and visualization, is accessible at https://github.com/Labo-MAB/Gagnon_FuzzyProfiles_2025.
